# Supplementary material for: Chromothripsis during telomere crisis is independent of NHEJ, and consistent with a replicative origin
Source: Genome Res. 2019 May;29(5):737–49. doi: 10.1101/gr.240705.118 (PMC6499312; doi:10.1101/gr.240705.118)
Supplement: Supplemental Material [file supp_gr.240705.118_Supplemental_file_1.zip › contigs/annotated_contigs/DB68/contig.3.DB68_length_560_mean_cov_7.91607142857.docx]

**DB68_length_560_mean_cov_7.91607142857**

```
AATCAGGGAAATGCAAATCAAACTACAATGAAATACTACTTCATTTAGAATGGCTATTAACAACAAGATAACTGTTAGCGAGGATGTGG
 >chr3:189575552-189575912 + E=5e-205
AGAGAAGAGAACCTTTACATACTTTCAGTGGGAATGTAAATTAGTACAGCCATTATGGAAAATAGTATAGAAGTTCCTTGAAAAATTAA

AAATATAACTACCATGTGATCCAGCAATCTCACTACTAGGCATTTATCCAAAGGGAATAAAATCAGTATGTCAGAGGGATATCTTCACT

CCTGTTTATTGCAGCACTATTTGCAATAGCCAAGATATGCAATCAACCTAAGTGTCCATCAAAGGATAAATGGATAAGAAAATGTTGTG

TATA|TGCACAGT|AGAATACTATTCAGTCAGAAAAACATAATGAAATCCTG|AGGTTGGGCAT|AGTGGCTCATGCCTGTAATCCCAG
             >chr3:50803521-50803559 - E=4e-02                >chr17:46563381-4656352
TACTTTGGGAGGCTGAGGCAGGAGGATCACCTTAGGTCAAGAGTTCGAGGCCTGGCCAACATGGCGAAACCCTGTCTCTACCAAAAATA
4 + E=2e-65
CAAAAATTAGCTGGGCATGGTGGTAGGCAC
```
